# Supplementary material for: Distinguishability of keystroke dynamic template
Source: PLoS One. 2022 Jan 21;17(1):e0261291. doi: 10.1371/journal.pone.0261291 (PMC8782503; doi:10.1371/journal.pone.0261291)
Supplement: Attachment — Submitted filename: Response to reviewer.pdf [file pone.0261291.s001.pdf]

Response to reviewers
Journal: PLOS ONE
Title: Distinguishability of Keystroke Dynamic Template
Paper ID: PONE-D-21-28587
==
Dear Editor,

We are grateful for their critical assessment of our work. These comments have helped us improve our work. We have revised our work according to the recommendation. The concerns are addressed point by point as the following.

Please include the following items when submitting your revised manuscript:

- A rebuttal letter that responds to each point raised by the academic editor and reviewer(s). You should upload this letter as a separate file labeled 'Response to Reviewers'.
- A marked-up copy of your manuscript that highlights changes made to the original version. You should upload this as a separate file labeled 'Revised Manuscript with Track Changes'.
- An unmarked version of your revised paper without tracked changes. You should upload this as a separate file labeled 'Manuscript'.

When submitting your revision, we need you to address these additional requirements.

1. Please ensure that your manuscript meets PLOS ONE's style requirements, including those for file naming. The PLOS ONE style templates can be found at

https://journals.plos.org/plosone/s/file?id=wjVg/PLOSOne_formatting_sample_main_body.pdf and

https://journals.plos.org/plosone/s/file?id=ba62/PLOSOne_formatting_sample_title_authors_affiliations.pdf

This has been done in the revision version.

2. We suggest you thoroughly copyedit your manuscript for language usage, spelling, and grammar. If you do not know anyone who can help you do this, you may wish to consider employing a professional scientific editing service. Whilst you may use any professional scientific editing service of your choice, PLOS has partnered with both American Journal Experts (AJE) and Editage to provide discounted services to PLOS authors. Both organizations have experience helping authors meet PLOS guidelines and can provide language editing, translation, manuscript formatting, and figure formatting to ensure your manuscript meets our submission guidelines. To take advantage of our partnership with AJE, visit the AJE website (http://learn.aje.com/plos/) for a 15% discount off AJE services. To take advantage of our partnership with Editage, visit the Editage website (www.editage.com) and enter referral code

PLOSEDIT for a 15% discount off Editage services. If the PLOS editorial team finds any language issues in text that either AJE or Editage has edited, the service provider will re-edit the text for free.
Upon resubmission, please provide the following:
The name of the colleague or the details of the professional service that edited your manuscript
A copy of your manuscript showing your changes by either highlighting them or using track changes (uploaded as a *supporting information* file)
A clean copy of the edited manuscript (uploaded as the new *manuscript* file)”

We have carefully corrected all the typos, grammar, and spelling in this revised version. This version has also been reviewed and edited by Dr. Nida Chatwattanasiri, National Electronics and Computer Technology Center (NECTEC), Thailand.

3. Thank you for stating the following in the Acknowledgments Section of your manuscript:

"This work was supported by the grant from National Science and Technology Development Agency (Grant No. FDA-CO-2562-9854-TH). "

We note that you have provided funding information that is not currently declared in your Funding Statement. However, funding information should not appear in the Acknowledgments section or other areas of your manuscript. We will only publish funding information present in the Funding Statement section of the online submission form. Please remove any funding-related text from the manuscript and let us know how you would like to update your Funding Statement. Currently, your Funding Statement reads as follows:

"NS
FDA-CO-2562-9854-TH
National Science and Technology 313
Development Agency
https://www.nstda.or.th/
The funders had no role in study design, data collection and analysis, decision to publish, or preparation of the manuscript"

Please include your amended statements within your cover letter; we will change the online submission form on your behalf.

As per funding statement, we would like to confirm that the following statement is accurate. In addition, we have removed funding related statement from the Acknowledgments section.
"NS
FDA-CO-2562-9854-TH
National Science and Technology 313
Development Agency
https://www.nstda.or.th/
The funders had no role in study design, data collection and analysis, decision to publish, or preparation of the manuscript"

============Reviewer 1==============================

Reviewer #1:

1. Abstract is not able to convey what is the technical contribution of this paper. I suggest to re-write it.

   Thank you for your comment. The following statements have been added to the abstract to describe the technical contribution of this paper.

   "… That is, in behavioral biometric verification, the user's templates are generally constructed using multiple enrolled samples to capture intra-user variation. This variation is then used to normalize the distance between a set of enrolled samples and a test sample. Then a normalized distance is compared against a predefined threshold value to derive a verification decision. As a result, the coverage area for accepted samples in the original space of vector representation is discrete. Therefore, users with the higher intra-user variation suffer higher false acceptance rates (FAR). This paper proposes a metric that can be used to reflect the verification performance of individual keystroke dynamic templates in terms of FAR. Specifically, the metric is derived from statistical information of user-specific feature variations, and it has a non-decreasing property when a new feature is added to a template…."

2. Improve the quality of figures and explain those properly.

   Thank you for your feedback. All the figures have been reworked to improve the quality and all the descriptions have been revised to improve the clarity. The changes are highlighted in blue color in the revised manuscript.

3. Result section is weak and I suggest authors to add more results and compare those with the existing approaches.

   Thank you for your comment. We have included a new subsection (5.3.4 Performance comparison to existing approaches) to discuss the verification performance comparison to existing work. Specifically, Table 5 Verification performance comparison of existing keystroke dynamic Approaches and Table 6 Entropy measures of different authentication factors have been added to compare verification performance of our work with existing keystroke dynamic approaches and to compare the entropy of keystroke dynamic with other authentication factors, respectively.

5.3.4 Performance comparison to existing approaches

In this subsection, the efficacy of the proposed quality metric for keystroke dynamic templates when compared to existing keystroke dynamic approaches is demonstrated. In addition, the security of keystroke dynamic in comparison to other authentication factors is evaluated. The performance result of the algorithm used in this paper is reported in Table 5 along with that of existing work. Note that, we have followed the protocol described in [8] to evaluate the overall performance of the algorithm used in this work when compared to existing approaches. That is, the threshold is set individually for each subject and the imposter samples for each subject were from the first five samples of all other subjects. While the reported performance using this protocol can be viewed as the upper bound performance of each algorithm, one challenging issue to be solved when the system is to be used in practical applications is how to set a threshold for each subject to achieve that level of performance. Nevertheless, this result demonstrates that the verification algorithm used in this work is comparable to other existing work when the same dataset and validation protocol is used. In addition, it confirms that the proposed metric can be used to measure quality of the keystroke dynamic template regardless of whether local or global thresholding approaches are used.

Lastly, entropy is another important metric to assess the security level of the system. In Table 6, the entropy measures of different authentication factors are reported. With this information, password-based two-factor authentication is estimated at 23.48-26.48 bits. Note that, the biometric entropy reported in this table is the relative entropy computed from KL divergence between genuine and imposter score distributions [28]. While the entropy of the keystroke dynamic is lower than that of the iris and fingerprint, it offers additional security at no user and hardware cost. That is, users are not required to perform additional tasks and user devices are required to equip with additional hardware.

Table 5. Verification performance comparison of existing keystroke dynamic approaches

Ref.	Dataset	Number of users	Keystroke length	Algorithm	EER (%)
[25]	SUNY	75	10	CNN plus RNN	9.75
[26]	CMU	51	10	Dependence Clustering	7.70
[27]	CMU	51	10	Nearest Neighborg	8.40
[8]	CMU	51	10	Manhattan (scaled with average absolute deviation)	9.60
This work	CMU	51	10	Manhattan (scaled with standard deviation)	9.16
				-Good users	4.50
				-Mid users	10.27
				-bad users	12.70

Table 6. Entropy measures of different authentication factors

Ref.	Authentication factor	Dataset description	Entropy (bits)
[29]	4-digits PIN	Dodonew, CSDN, Rockyou, Yahoo (total 3.4 M)	8.41
[29]	6-digits PIN	Dodonew, CSDN, Rockyou, Yahoo (total 6.4 M)	13.21
[30]	Password	14 datasets (total 113.3 M)	20-23
[28]	Iris	ICE (High quality set - 374 iris, 10 samples each)	8.9-10 8.9-10 bits
[31]	Finger Vein	VERA (220 fingers, 2 samples each) UTFVP (360 fingers, 4 images each)	4.2 - 13.2 18.9-19.5
This work	Keystroke	CMU (51 typists, 400 samples each) -Good user	3.48 4.62 bits

4. Although this paper is well written, there are still some typos in the current version. I would like to suggest the authors carefully proofread this paper and correct all the typos in the revision.

   Thank you very much for your suggestion. We have carefully proofread the paper and correct all the typos and spelling. We hope it now matches the journal standard.

5. Some very related and recent work may be discussed to improve the quality of literature. e.g.

   - Efficient and secure routing protocol based on artificial intelligence algorithms with UAV-assisted for vehicular Ad Hoc networks in intelligent transportation systems
   - An overview of Internet of Things (IoT): Architectural aspects, challenges, and protocols
   - Security in Internet of Things: issues, challenges, taxonomy, and architecture
   - Improving Road Safety for Driver Malaise and Sleepiness Behind the Wheel Using Vehicular Cloud Computing and Body Area Networks,
   - A trust infrastructure based authentication method for clustered vehicular ad hoc networks
   - The optimal path finding algorithm based on reinforcement learning
   - The Effect of Gender, Age, and Education on the Adoption of Mobile Government Services
   - IoT-based Big Data secure management in the Fog over a 6G Wireless Network,
     Using vehicles as fog infrastructures for transportation cyber-physical systems (T-CPS): Fog computing for vehicular networks

   Thank you for your comment. We have included more discussion on applications of keystroke dynamic in section 2 Related work. In addition, more reference work has been cited. Details are as follows.

   2 Related work

As the number of approaches for user authentication has been proposed as an alternative to password, Bonneau et al. [16] has proposed a framework to compare and contrast those authentication schemes based on three main factors: usability, deployability, and security. In addition, the study revealed that, while traditional biometric-based authentication schemes (fingerprint, iris, voice) have some advantages over password based authentication in terms of usability (memorability, scalable-for-user, and physically-effortless) and security (physical observation and throttled-Guessing), its deployability is the main setback for the schemes as it is not server and browser compatible. As such, the current use is limited to on-device authentication such as unlocking the phone or authorizing device access to use services. Consequently, the password still plays an important role for general-purpose user authentication mechanism. On the other hand, many proposals to resolve these issues of the original password authentication scheme have been proposed. For example, password managers that require the users to log in with their master password can be used to resolve usability in terms of memorability, scalable-for-user, physically-effortless, physical observation and but fail to resolve security in terms of physical observation and throttled-guessing. In this case, behavioral biometric gleaned from keystroke information can be used to provide resilience to physical observation and throttled-guessing. In addition, Qiu et al. [17] has proposed a secure three-factor authentication protocol where all three types of authentication factors are provided by a user. Moreover, Jiang et al. [18] has proposed another secure three-factor remote authentication protocol to preserve privacy of biometric information. These proposals allow behavioral biometric gleaned from keystroke information to be used in remote setting thereby enhancing deployability of password-based two factor authentication (password string and typing pattern). As such, the mechanism can also be used for user-IoT devices authentication where data and services on these devices are becoming more sensitive and the need for secure authentication mechanism is increasing [19,20].

[16]. Bonneau J, Herley C, Van Oorschot PC, Stajano F. The quest to replace passwords: A framework for comparative evaluation of web authentication schemes. In: 2012 IEEE Symposium on Security and Privacy. IEEE; 2012. p. 553–567.

[17]. Qiu S, Wang D, Xu G, Kumari S. Practical and provably secure three-factor authentication protocol based on extended chaotic-maps for mobile lightweight devices. IEEE Transactions on Dependable and Secure Computing. 2020

[18]. Jiang Q, Zhang N, Ni J, Ma J, Ma X, Choo KKR. Unified biometric privacy preserving three-factor authentication and key agreement for cloud-assisted autonomous vehicles. IEEE Transactions on Vehicular Technology. 2020;69(9):9390–9401. 442

[19]. Gupta BB, Quamara M. An overview of Internet of Things (IoT): Architectural aspects, challenges, and protocols. Concurrency and Computation: Practice and Experience. 2020;32(21):e4946.

[20]. Dhillon PK, Kalra S. A lightweight biometrics based remote user authentication scheme for IoT services. Journal of Information Security and Applications. 2017;34:255–270.

6. The authors are expected to report the running time of the proposed algorithm in the revision.

   We have included this information at the beginning of section 5.3 Result as the following.

   "... Note that, computational overhead for deriving distinctiveness score is O(n) where n is the number of features used to compute the metric (or the number of input characters). It took 0.127 milliseconds on average to compute the distinctiveness from a set of keystroke samples for each template with 10 characters (or 0.0127 milliseconds per character) using Matlab R2019b running on Windows 10 with AMD Ryzen 7 3800x. …"

7. As an additional remark, references need to be completed with all the required information (e.g. page number, name of journal/conference, vol., issues, etc).
   Based on the comments above, I would like to accept this paper if my following concerns are carefully addressed.

   We have carefully reviewed the details of references and revised them accordingly. Thank you very much for your valuable feedback that help us improve the quality of this paper.

===========Reviewer 2===============================

Reviewer #2: This paper addresses the issue of varied security level of Keystroke Dynamic by proposing a metric to quantify the quality of keystroke dynamic templates. Specifically, the metric is derived from statistical information of user specific feature variations and it can be used to reflect the verification performance of individual keystroke dynamic template in terms of false acceptance rate (FAR). The results seem nice. Overall, I like this paper, yet there are some issues to be addressed before publication.

Strength:

1) The motivation and idea are good.

2) The comprison of different template groups of keystroke behavior based user authentication in Table is very nice.

3) The experimental results seem to be reseaonable and practical.

Weakness:

1. There is no rationale to explain why the proposed template/metric works. The authors shall use one or two key sentenses to explain why the new template/metric works better than existing schemes.

    Thank you for your comment. We have included the following statements to explain the concept of the proposed method.

    “Using the proposed metric, the template with lower intra-user variation between enrolled samples (lower σ) would have higher distinctiveness as the coverage area for accepted samples in the original space of vector representation would be smaller. In addition, the keystroke template with an additional typing character would always have a higher distinctiveness. This is an important property as the distinctiveness should never decrease when additional sequences are appended to the keystroke.”

2. Recently, there have been quite a number of new behavior authentication schemes proposed (as well as other kinds of authentication schemes like multi-factor authentication schemes). The authors shall compare the proposed new template with these existing schemes using a table like Table I of the IEEE S&P12 paper and Table IV of the IEEE TII'18 paper.

    - "The quest to replace passwords- a framework for comparative evaluation of Web authentication schemes", IEEE S&P 2012.
    - "Measuring Two-Factor Authentication Schemes for Real-Time Data Access in Industrial Wireless Sensor Networks." IEEE TII, 2018.
    - "Evaluating Behavioral Biometrics for Continuous Authentication： Challenges and Metrics", Proc. Asiaccs 2017.

    Thank you for your suggestion. We have included the discussion on this topic in the section 2 as follows.

2 Related work

As the number of approaches for user authentication has been proposed as an alternative to password, Bonneau et al. [16] has proposed a framework to compare and contrast those authentication schemes based on three main factors: usability, deployability, and security. In addition, the study revealed that, while traditional biometric-based authentication schemes (fingerprint, iris, voice) have some advantages over password based authentication in terms of usability (memorability, scalable-for-user, and physically-effortless) and security (physical observation and throttled-Guessing), its deployability is the main setback for the schemes as it is not server and browser compatible. As such, the current use is limited to on-device authentication such as unlocking the phone or authorizing device access to use services. Consequently, the password still plays an important role for general-purpose user authentication mechanism. On the other hand, many proposals to resolve these issues of the original password authentication scheme have been proposed. For example, password managers that require the users to log in with their master password can be used to resolve usability in terms of memorability, scalable-for-user, physically-effortless, physical observation and but fail to resolve security in terms of physical observation and throttled-guessing. In this case, behavioral biometric gleaned from keystroke information can be used to provide resilience to physical observation and throttled-guessing. In addition, Qiu et al. [17] has proposed a secure three-factor authentication protocol where all three types of authentication factors are provided by a user. Moreover, Jiang et al. [18] has proposed another secure three-factor remote authentication protocol to preserve privacy of biometric information. These proposals allow behavioral biometric gleaned from keystroke information to be used in remote setting thereby enhancing deployability of password-based two factor authentication (password string and typing pattern). As such, the mechanism can also be used for user-IoT devices authentication where data and services on these devices are becoming more sensitive and the need for secure authentication mechanism is increasing [19,20].

[16]. Bonneau J, Herley C, Van Oorschot PC, Stajano F. The quest to replace passwords: A framework for comparative evaluation of web authentication schemes. In: 2012 IEEE Symposium on Security and Privacy. IEEE; 2012. p. 553–567.

[17]. Qiu S, Wang D, Xu G, Kumari S. Practical and provably secure three-factor authentication protocol based on extended chaotic-maps for mobile lightweight devices. IEEE Transactions on Dependable and Secure Computing. 2020

[18]. Jiang Q, Zhang N, Ni J, Ma J, Ma X, Choo KKR. Unified biometric privacy preserving three-factor authentication and key agreement for cloud-assisted autonomous vehicles. IEEE Transactions on Vehicular Technology. 2020;69(9):9390–9401. 442

[19]. Gupta BB, Quamara M. An overview of Internet of Things (IoT): Architectural aspects, challenges, and protocols. Concurrency and Computation: Practice and Experience. 2020;32(21):e4946.

[20]. Dhillon PK, Kalra S. A lightweight biometrics based remote user authentication scheme for IoT services. Journal of Information Security and Applications. 2017;34:255–270.

3. The entropy (security) is also a key factor for biometric/behavior identification. I suggest the authors compare the entropy of keystroke with other authentication factors. Otherwise, it is difficult to show the superiority of the keystroke authentication factor. For instance, Sadeghi et. al. showed that EEG biometric entropy is at best 83-bits; Wang et al. showed that the entropy of human chosen 4-digit PINs is 8.41 bits and 6-digit PINs 13.21 bits; Feng et al. showed that the entropy of human face template is 75 bits; Wang et al. showed that the entropy of human chosen passwords is 20-22 bits; Inthavisas et al. showed that the entropy of voice is 18-30 bits.
   - Wang et al., Understanding Human-Chosen PINs: Characteristics, Distribution and Security. Proc. ACM ASIACCS 2017.
   - Sadeghi et al., Geometrical anal- ysis of machine learning security in biometric authentication systems, in:2017 16th IEEE International Conference on Machine Learning and Ap- plications (ICMLA), 2017
   - Feng et al., Binary discriminant analysis for generating binary face template, IEEE Transactions on Information Forensics and Security, 2012.
   Wang et al., Zipf's law in passwords, IEEE Transactions on Information Forensics and Security, 2017 2776–2791.
   - Inthavisas et al., Secure speech biometric templates for user authentication, IET Biometrics, 2012.

   Thank you for your suggestion. We have included a new subsection (5.3.4 Performance comparison to existing approaches) to discuss the verification performance comparison to existing work. Specifically, Table 5 Verification performance comparison of existing keystroke dynamic Approaches and Table 6 Entropy measures of different authentication factors have been added to compare verification performance of our work with existing keystroke dynamic approaches and to compare the entropy of keystroke dynamic with other authentication factors, respectively.

   5.3.4 Performance comparison to existing approaches

   In this subsection, the efficacy of the proposed quality metric for keystroke dynamic templates when compared to existing keystroke dynamic approaches is demonstrated. In addition, the security of keystroke dynamic in comparison to other authentication factors is evaluated. The performance result of the algorithm used in this paper is reported in Table 5 along with that of existing work. Note that, we have followed the protocol described in [8] to evaluate the overall performance of the algorithm used in this work when compared to existing approaches. That is, the threshold is set individually for each subject and the imposter samples for each subject were from the first five samples of all

other subjects. While the reported performance using this protocol can be viewed as the upper bound performance of each algorithm, one challenging issue to be solved when the system is to be used in practical applications is how to set a threshold for each subject to achieve that level of performance. Nevertheless, this result demonstrates that the verification algorithm used in this work is comparable to other existing work when the same dataset and validation protocol is used. In addition, it confirms that the proposed metric can be used to measure quality of the keystroke dynamic template regardless of whether local or global thresholding approaches are used.

Lastly, entropy is another important metric to assess the security level of the system. In Table 6, the entropy measures of different authentication factors are reported. With this information, password-based two-factor authentication is estimated at 23.48-26.48 bits. Note that, the biometric entropy reported in this table is the relative entropy computed from KL divergence between genuine and imposter score distributions [28]. While the entropy of the keystroke dynamic is lower than that of the iris and fingerprint, it offers additional security at no user and hardware cost. That is, users are not required to perform additional tasks and user devices are required to equip with additional hardware.

Table 5. Verification performance comparison of existing keystroke dynamic approaches

Ref.	Dataset	Number of users	Keystroke length	Algorithm	EER (%)
[25]	SUNY	75	10	CNN plus RNN	9.75
[26]	CMU	51	10	Dependence Clustering	7.70
[27]	CMU	51	10	Nearest Neighborg	8.40
[8]	CMU	51	10	Manhattan (scaled with average absolute deviation)	9.60
This work	CMU	51	10	Manhattan (scaled with standard deviation)	9.16
				-Good users	4.50
				-Mid users	10.27
				-bad users	12.70

Table 6. Entropy measures of different authentication factors

Ref.	Authentication factor	Dataset description	Entropy (bits)
[29]	4-digits PIN	Dodonew, CSDN, Rockyou, Yahoo (total 3.4 M)	8.41
[29]	6-digits PIN	Dodonew, CSDN, Rockyou, Yahoo (total 6.4 M)	13.21
[30]	Password	14 datasets (total 113.3 M)	20-23
[28]	Iris	ICE (High quality set - 374 iris, 10 samples each)	8.9-10<br>8.9-10 bits
[31]	Finger Vein	VERA (220 fingers, 2 samples each)<br>UTFVP (360 fingers, 4 images each)	4.2 - 13.2<br>18.9-19.5
This work	Keystroke	CMU (51 typists, 400 samples each)<br>-Good user	3.48<br>4.62 bits

4. Behavior based user authentication factors are generally not separately used as authentication, especially for behaviors that can be relatively easy recorded/leaked/stealed. Thus, they are often combined with tradtional authentication factors like passwords and PINs.
"Practical and Provably Secure Three-Factor Authentication Protocol Based on Extended Chaotic-Maps for Mobile Lightweight Devices", IEEE Transactions on Dependable and Secure Computing, 2020, Doi: 10.1109/TDSC.2020.3022797
"Unified Biometric Privacy Preserving Three-factor Authentication and Key Agreement for Cloud-assisted Autonomous Vehicles".?IEEE Transactions on Vehicular Technology. DOI: 10.1109/TVT.2020.297125
"Two Birds with One Stone: Two-Factor Authentication with Security Beyond Conventional Bound." IEEE TDSC 2018.

Thank you very much for your insightful comment. The discussion on this topic has also been included in the new section "Related work"

"In addition, Qiu et al. [17] has proposed a secure three-factor authentication protocol where all three types of authentication factors are provided by a user. Moreover, Jiang et al. [18] has proposed another secure three-factor remote authentication protocol to preserve privacy of biometric information. These proposals allow behavioral biometric gleaned from keystroke information to be used in remote setting thereby enhancing deployability of password-based two factor authentication (password string and typing pattern). As such, the mechanism can also be used for user-IoT devices authentication where data and services on these devices are becoming more sensitive and the need for secure authentication mechanism is increasing [19,20]."
